# Non-Invasive Brain Stimulation: A New Strategy in Mild Cognitive Impairment?

**DOI:** 10.3389/fnagi.2017.00016

**Published:** 2017-02-13

**Authors:** Agustina Birba, Agustín Ibáñez, Lucas Sedeño, Jesica Ferrari, Adolfo M. García, Máximo Zimerman

**Affiliations:** ^1^Laboratory of Experimental Psychology and Neuroscience (LPEN), Institute of Cognitive and Translational Neuroscience (INCyT), INECO Foundation, Favaloro UniversityBuenos Aires, Argentina; ^2^National Scientific and Technical Research Council (CONICET)Buenos Aires, Argentina; ^3^Universidad Autónoma del CaribeBarranquilla, Colombia; ^4^Center for Social and Cognitive Neuroscience (CSCN), School of Psychology, Universidad Adolfo IbañezSantiago de Chile, Chile; ^5^Centre of Excellence in Cognition and its Disorders, Australian Research Council (ARC)Sydney, NSW, Australia; ^6^Faculty of Education, National University of Cuyo (UNCuyo)Mendoza, Argentina

**Keywords:** mild cognitive impairment, non-invasive brain stimulation, neuroenhancement, transcranial magnetic stimulation, transcranial direct current stimulation

## Abstract

Non-invasive brain stimulation (NIBS) techniques can significantly modulate cognitive functions in healthy subjects and patients with neuropsychiatric disorders. Recently, they have been applied in patients with mild cognitive impairment (MCI) and subjective cognitive impairment (SCI) to prevent or delay the development of Alzheimer’s disease (AD). Here we review this emerging empirical corpus and discuss therapeutic effects of NIBS on several target functions (e.g., memory for face-name associations and non-verbal recognition, attention, psychomotor speed, everyday memory). Available studies have yielded mixed results, possibly due to differences among their tasks, designs, and samples, let alone the latter’s small sizes. Thus, the impact of NIBS on cognitive performance in MCI and SCI remains to be determined. To foster progress in this direction, we outline methodological approaches that could improve the efficacy and specificity of NIBS in both conditions. Furthermore, we discuss the need for multicenter studies, accurate diagnosis, and longitudinal approaches combining NIBS with specific training regimes. These tenets could cement biomedical developments supporting new treatments for MCI and preventive therapies for AD.

## Introduction

In the face of an aging society, an urgent need has emerged to prevent and treat dementia, in general, and Alzheimer’s disease (AD), in particular (Ferri et al., 2006). Despite wide-ranging research efforts to elucidate the putative pathological mechanisms of dementia, available treatments remain limited (Alzheimer’s Association, 2012). Promisingly, however, various factors have been shown to boost the dynamics of relevant neural networks, delaying and even reducing clinical symptoms (Fregni and Pascual-Leone, 2007). Therefore, interventions that enhance neuroplastic responses in early disease stages might be particularly useful to attenuate the rate of cognitive decline in AD patients.

A key model to assess this possibility is afforded by patients with mild cognitive impairment (MCI). This construct has evolved over the past two decades to denote a cognitive state intermediate between normal aging and very early dementia (Petersen and Negash, 2008). Patients with this condition feature objective memory impairment and other cognitive deficits, but their dysfunction is not so severe as to compromise daily activities (Petersen and Negash, 2008). MCI comprises four clinical subtypes: (i) single-domain amnestic MCI (aMCI); (ii) multiple-domain aMCI; (iii) single-domain non-amnestic MCI; and (iv) multiple-domain non-amnestic MCI (Winblad et al., 2004; Anstey et al., 2008; Albert et al., 2011). These subtypes differ in etiology and outcome: whereas both forms of aMCI imply a high chance of progression to AD, non-amnestic varieties involve considerable risk of conversion to non-AD dementia (Winblad et al., 2004; Anstey et al., 2008; Albert et al., 2011).

A second relevant model can be found in individuals featuring subjective cognitive impairment (SCI). This notion encompasses people who report everyday concerns about their cognitive functioning, even in the absence of objectively established impairments. Given its association with subsequent cognitive decline, SCI represents a potential prodromal state of AD occurring even before MCI (Reisberg and Gauthier, 2008).

In sum, aMCI and SCI are likely to anticipate incipient dementia. Despite the high between-patient variability and the uncertainty about their causative physiopathological mechanisms, both conditions offer highly relevant models to implement interventions aimed to boost cognitive performance through modulations of neuronal activity and connectivity. In this sense, non-invasive brain stimulation (NIBS) techniques have emerged as a potentially useful alternative. These tools can selectively enhance adaptive activity patterns, suppress maladaptive patterns, and influence learning and skill acquisition processes by inducing synaptic plasticity and network reorganization (Hummel and Cohen, 2006; Nitsche et al., 2008). In particular, NIBS can have a direct impact on memory mechanisms (including working, episodic, and associative memory) in young adults, elderly adults and patients with neurological dysfunctions (Manenti et al., 2012; Elder and Taylor, 2014).

Building on these antecedentes, a number of studies have assessed the impact of NIBS on MCI patients. Here we aim to critically review such emerging literature. While other reviews have addressed the impact of NIBS in cognitively impaired patients (Freitas et al., 2011; Nardone et al., 2014), here we focus on the key questions and difficulties of setting a treatment protocol. To this end, first we characterize the main features of NIBS. Thereupon, we review available studies examining the impact of NIBS on learning and memory in MCI and SCI. In particular, we describe and discuss in detail the studies’ protocols and parameters, highlight the advantages of combining NIBS with cognitive training. Finally, we outline methodological strategies that could promote major developments in this promising research area.

## NIBS: Features and Mechanisms

With NIBS, researchers can transiently influence behavior by altering neural activity (Miniussi et al., 2013). A considerable body of evidence has been forged through the two most commonly used techniques in humans: repetitive transcranial magnetic stimulation (rTMS) and transcranial direct current stimulation (tDCS). Although they differ in several aspects (Table 1), both tools can induce long-term after effects on cortical excitability and neuronal plasticity. Typically, this brings about facilitatory or inhibitory effects (Nitsche and Paulus, 2000), broadly mirroring the workings of long-term potentiation (LTP) and long-term depression (LTD; Dayan et al., 2013). Since such effects can considerably outlast the actual stimulation period, NIBS techniques open valuable avenues for rehabilitation, especially when stimulation protocols are combined with adequate training-based interventions (Zimerman et al., 2013).

**Table 1:** **Summary of the main features of non-invasive brain stimulation (NIBS) techniques**.

	**TMS**	**tDCS**
Excitatory	High frequency >5 Hz	Anodal stimulation
Inhibitory	Low frequency ~1 Hz	Cathodal stimulation
Mechanism of action	Neuronal depolarization	Membrane modulation
Physiological substrate	NMDA receptor	Voltage-dependent sodium and calcium channels; NMDA receptor
Focality of stimulation	More focal	Less focal
Design of sham-controlled double-blind studies	More difficult	Less difficult
Synchronous application with specific training	More difficult	Less difficult
Discomfort or pain	Mild following sustained application	Mild at the beginning
Adverse effects	Rare, if applied in accordance with safety guidelines	Rare, if applied in accordance with safety guidelines. Sensory discomfort, occasional headaches, no seizures described
Time resolution	Excellent: milliseconds	Poor
Cost	Higher	Lower
Simultaneous combination with EEG	Possible, but with several TMS-related artifacts	Possible. However, brain activity cannot be synchronously recorded from the same channels used for stimulation
Simultaneous combination with fMRI	Possible	Possible, but with electrode artifacts and the remote risk of sudden electrode heating
Portability	Not portable	Portable

*Modified from Zimerman and Hummel (2010)*.

TMS has been recently approved in some countries to treat depression and other disorders, subject to approval by local ethical review boards (Rossi et al., 2009). These reservations largely follow from the suboptimal understanding of the putative mechanisms underlying NIBS-related effects. However, a number of contributing factors have been recently identified. A critical one concerns modulation of neurotransmitter levels (Ridding and Ziemann, 2010). Transcranial stimulation may alter the dynamics of excitatory/inhibitory neurotransmitter systems (e.g., GABA and glutamate) affecting the regulation of cortical neuronal activity. For example, rTMS effects decrease if the subjects consume a drug that interferes with NMDA receptors (Reis et al., 2006). Likewise, post-tDCS effects can be blocked by an NMDA-antagonist, whereas a partial NMDA-agonist (d-cycloserine) can selectively extend the duration of motor cortical excitability induced by anodal tDCS (Nitsche et al., 2004). Moreover, MR spectroscopy research (Stagg et al., 2009) shows that anodal tDCS decreases GABAergic transmission, while cathodal tDCS yields similar effects on glutamate concentrations over the motor cortex (MC).

NIBS may also exert long-lasting plastic changes through gene induction. Research based on rTMS protocols (Fritsch et al., 2010) has shown significant enhancements of brain-derived neurotrophic factor (BDNF) mRNA in the hippocampal, parietal, and piriform cortices. For instance, LTP-like effects in adult mice are abolished for specimens with deletion of the BDNF gene, suggesting that this factor could be a critical mediator of direct stimulation effects (Fritsch et al., 2010). Importantly, as a member of the neurotrophin family, BDNF plays an important role in synaptogenesis and synaptic mechanisms related to learning and memory processes.

Additionally, TMS has been successfully used in AD and MCI to track and modulate abnormal mechanisms, including glutamatergic dysfunctions, disruption of synaptic plasticity, and inhibition of LTP (Nardone et al., 2014). For example, TMS research consistently shows that such patients feature a decreased resting motor threshold, which has been interpreted as the electrophysiological correlate of cortical glutamatergic dysfunctions and as a marker of reduced cortical hyperexcitability in AD (Di Lazzaro et al., 2003, 2004; Inghilleri et al., 2006; Trebbastoni et al., 2016). Furthermore, rTMS reduces facilitation of the motor-evoked potential (MEP) in aMCI patients (Trebbastoni et al., 2016), revealing an initial impairment of the mechanisms that underlie glutamate-induced synaptic potentiation (Trebbastoni et al., 2016). On the other hand, a TMS-MRI study indicates that hyperexcitability of the sensorimotor cortex might represent a protective mechanism counteracting the prominent loss of cortical volume in AD and MCI. However, this supposed protective mechanism was found neither on the precuneus or cuneus of AD patients, nor in an MCI group (Niskanen et al., 2011). Furthermore, an assessment of navigated TMS-evoked EEG responses in AD patients showed prominent changes in functional connectivity and reactivity (Julkunen et al., 2008, 2011). In particular, TMS-evoked responses at 30–50 ms decreased significantly in AD over widespread brain regions, suggesting dysfunctions of a large-scale sensorimotor network. Compatibly, a TMS-EEG co-registration study in AD patients offered direct evidence of MC hyperexcitability, extending to the whole sensorimotor system (Ferreri et al., 2016). Although speculative, these changes could be interpreted as a compensatory mechanism allowing for the preservation of sensorimotor programming and execution over a long period, regardless of the disease’s progression. Additional evidence on cortical plasticity in relevant patient samples comes from paired associative stimulation (PAS) and cortical responses to rTMS. Null PAS effects in MCI patients have been reported by Lahr et al. (2016). However, this result should be considered with caution, since the study mixed patients with single- and multiple-domain aMCI which may have clouded effects on a specific subsample. Indeed, previous evidence from Nardone et al. (2012) demonstrated that short-latency afferent inhibition is significantly reduced in patients with multiple-domain amnestic MCI relative to controls, but not in patients with single-domain amnestic MCI or with non-amnestic MCI. Taken together, this incipient evidence suggests that NIBS can directly impinge on the pathological mechanisms underlying some forms of AD and MCI.

Finally, note that the effects of rTMS and tDCS are not restricted to the stimulation site. Depending on stimulation parameters, they can induce changes at distant points through effective modulations of remote, interconnected networks (Plewnia et al., 2003; Hummel and Cohen, 2006). This particular property of NIBS techniques has been assessed in proof-of-principle studies aimed to enhance and decrease excitability of critical brain regions involved in the recovery process (for details see Hummel and Cohen, 2006; Nowak et al., 2010; Schulz et al., 2013). All these tenets illuminate the evidence gathered so far in NIBS studies on MCI patients, which we review below.

## Does NIBS Improve Learning and Formation of Novel Memories in MCI?

Growing evidence indicates that NIBS techniques can significantly modulate various cognitive processes in both elderly neurotypicals and patients with AD. While the impact of NIBS on the latter population has been widely discussed (Hsu et al., 2015), much less attention has been paid to NIBS research on MCI and SCI. Below we offer a joint assessment of this empirical corpus, discussing available results in terms of stimulated brain regions. We deal with TMS studies first, and then consider those employing tDCS (for full details of each study see Table 2).

**Table 2:** **Summary of studies using NIBS to influence memory function in MCI patients**.

Experiment	Participants	NIBS	Study design	Stimulation protocol	Target area	Stimulated cognitive process and outcome	Control condition	Tests/ follow-up	Results
Anderkova et al. (2015)	8 MCI and 12 AD patients Age: 73 ± 7	rTMS	Crossover design. Each patient received one stimulation session in a random order, over each target area	Three 22-min sessions at 10 Hz. (1/day)	IFG-STG	Sustained attention, psychomotor speed, processing efficacy, set-switching, executive functions, processing speed, attentional and visual processing, encoding and recognition	VTX	Prior and immediately after each session	Enhanced speed processing
Eliasova et al. (2014)	10 aMCI patient Age: 72 ± 8	rTMS	Cross Overdesign. Each patient received one stimulation sessions in a random order, over each target area	Two 22-min sessions at 10 Hz. (1/day)	IFG	Sustained attention, psychomotor speed, efficacy of cognitive processing, set-switching, executive functions, speed cognitive process attentional and visual processing, encoding and recognition	VTX	Prior and immediately after each session.	improvement of attention and psychomotor speed
Cotelli et al. (2012)	1 aMCI patient Age: 69 22 healthy controls Age: 64 ± 4	rTMS	Uncontrolled *pre-post* study	Ten 25-min sessions at 20 Hz (5/week)	IPL	Associative memory, reasoning language, learning, short and long term memory, praxis attention, executive functions	Uncontrolled	2 baselines, immediately after stimulation protocol (2 weeks), 24 weeks	Enhanced associative memory, long term memory
Drumond Marra et al. (2015)	34 MCI patients Age: 60–74	rTMS	Double blind controlled study	Ten 25-min sessions at 10 HZ (5/week)	Left DLPFC	Cognitive processing, everyday memory, logical memory, long-term narrative memory, short-term auditory-verbal memory, rate of learning, learning and retrieval, working memory, executive functions	Sham group with a placebo coil.	Immediately and 30 days after stimulation protocol	Improved everyday memory for at least 1 month
Meinzer et al. (2015)	18 MCI patients Age: 69.56 ± 5.56 18 healthy controls Age: 67.44 ± 7.27	atDCS	Double-blind, cross-over, randomized, sham stimulation. During Cognitive Task and resting state fMRI	One session at 1 mA during 20 min.	Left ventral IFG	Cognitive processing.	Sham group: atDCS turned off after 30 s	During stimulation No follow-up	Improved word retrieval. Reduced activity in the bilateral prefrontal cortex, right middle temporal gyrus, left basal ganglia and thalamus
Sedlackova et al. (2008)	7 MCI-V patients Age: 70.3 ± 8.7	rTMS	Randomized, blind, cross-over study	One 30-min session at 10 HZ (1/day)	Left DLPFC	Executive function, working memory, and psychomotor speed.	MC (control site)	During stimulationNo follow-up	No effects
Turriziani et al. (2012)	8 MCI patients 66.4 ± 5.7 100 healthy controls Age: 20–35	rTMS	Blind, crossover study	One 10-min session at 1 Hz.	Left and right DLPFC	Cognitive processing, non-verbal recognition memory, verbal recognition memory	Sham group: coil held close to the DLPFC but angled away	Immediately after stimulation. No follow-up	Improved the performance on the non-verbal recognition memory test
Solé-Padullés et al. (2006)	40 adults with SCI Age: 66.95 ± 9.43	rTMS	Pre-post fMRI, randomized double blind sham stimulation	One 5-min session at 5 Hz	PFC	Associative memory	Sham group: coil held tangentially to the head, with its edge resting on the scalp.	Immediately after stimulation during post fMRI	Improvement in associative memory task. Higher activation of the right inferior and middle frontal giry together with the middle and superior occipital gyri.
Manenti et al. (2016)	20 PD patients with MCI Age: 67.1 ± 7.2	atDCS	Double-blind, cross-over, randomized, sham stimulation. During physical therapy.	One session at 2 mA during 25 min per (5/week)	DLPFC	Motor physical therapist for PD. Outcome evaluated: clinical neuropsychological, and motor task,	Sham group: atDCS turned off after 30 s	Prior and immediately after and 3 month follow up	Improvement in motor abilities, reduction of depressive symptoms in sham and atDCS. Improvement in the PD cognitive Rating Scale and verbal fluency test, only in tDCS.

*NIBS, Non-invasive brain stimulation; atDCS, anodal transcranial direct current stimulation; rTMS, repetitive transcranial magnetic stimulation; AD, Alzheimer’s disease; MCI, mild cognitive impairment; VMCI, vascular mild cognitive impairment; PD, Parkinson’s disease; SCI, subjective cognitive impairment; DLPFC, dorsolateral prefrontal cortex; IFG, inferior frontal gyrus; IPL, inferior parietal lobule; MC, motor cortex; PFC, prefrontal cortex; STG, superior temporal gyrus; VTX, vertex*.

### TMS Studies with MCI Patients

#### Prefrontal Cortex

In a study with SCI patients, Solé-Padullés et al. (2006) reported improvements of face-name associative memory following up-regulation of the bilateral prefrontal cortex (PFC) through high-frequency (5 Hz) rTMS. Specifically, the behavioral enhancement positively correlated with the recruitment of the right PFC and posterior bilateral cortices, as shown by fMRI. In another study, Turriziani et al. (2012) found improvements of non-verbal recognition memory in MCI patients after low-frequency (1 Hz) stimulation and subsequent down-regulation of the right but not the left dorsolateral prefrontal cortex (DLPFC). Morever, a double-blind randomized sham-controlled study using 10-Hz rTMS in MCI patients (Drumond Marra et al., 2015) showed that up-regulation of the DLPFC led to improvements in everyday memory. Interestingly, this enhancement remained for at least a month. However, stimulation of prefrontal structures has also yielded null results. In particular, a blind study with a crossover design (Sedlackova et al., 2008) failed to induce positive or negative behavioral effects following either low- or high-frequency rTMSof the DLPFC in seven patients with vascular MCI.

#### Parietal Cortex

In a single-case study with an aMCI patient, Cotelli et al. (2012) assessed whether up-regulation of the left parietal cortex could lead to long-term improvements in face-name associations. The 10-day protocol consisted in daily 25-min-long stimulation sessions with high-frequency (20 Hz) rTMS. Behavioral enhancements suggested a putative role of the target region in associative memory.

#### Inferior Frontal Gyrus and Superior Temporal Gyrus

Two related studies Eliasova et al. (2014) and Anderkova et al. (2015) found that rTMS significantly improved Stroop-task performance in aMCI and AD patients. The stimulation protocol comprised three randomized 22-min-long sessions, during which 10-Hz rTMS was applied over the IFG, the right superior temporal gyrus (STG), and the vertex (VTX, a control stimulation site). As compared to the VTX, both IFG and STG simulation improved the patients’ behavioral outcome. In particular, through a combination of rTMS and MRI, Anderkova et al. (2015) found that MCI/AD subjects had significant atrophy in the inferior temporal and fusiform gyri, putamen and cerebellum relative to healthy controls. Moreover, the level of atrophy correlated with the change in Stroop-task performance after rTMS of the STG.

#### Interim Summary

Taken together, these studies indicate that rTMS can have significant effects on cognitive functions in patients with SCI and MCI. Specifically, this form of stimulation appears to enhance face-name association memory, non-verbal recognition memory, attention, psychomotor speed, and everyday memory. However, inconsistencies among comparable studies and null results in some of them cast doubts on the robustness of this technique to restore cognitive function in MCI. A number of caveats can be identified. First, rTMS might not directly stimulate deep brain areas critically compromised in MCI and AD (e.g., the hippocampus). Second, discrepancies in stimulation parameters (frequency, intensity, duration, number of sessions, stimulation site) and experimental design across studies make it difficult to determine which configuration could systematically yield effective results. Finally, there is only one double-blind randomized sham-controlled rTMS study with MCI patients. Thus, further work and more randomized placebo-controlled studies are needed to establish the optimal stimulation parameters for potential treatments in this population. Critically, a more through characterization is needed of how results are influenced by multiple interrelated factors, such as the patients’ clinical profile, specific target areas, stimulation frequency, number of treatment sessions, sessionduration and specific cognitive domains tapped through selected behavioral tasks.

### tDCS Studies with MCI Patients

To date, only two studies have explored the potential neurocognitive benefits of anodal tDCS in MCI patients. The target regions in them were the IFG and the PFC.

#### Inferior Frontal Gyrus

Meinzer et al. (2015) performed a double-blind, crossover, sham-controlled stimulation study with simultaneous task-related and resting-state fMRI recordings. An MRI-compatible stimulator was used to administer a constant direct current (1 mA, for 20 min) over the IFG. During sham stimulation, MCI patients produced fewer correct responses than healthy controls in a semantic fluency task. Interestingly, the degree of correct responses was associated with hyperactivity in bilateral prefrontal regions. Anodal tDCS significantly improved performance in MCI patients to healthy-control levels. Furthermore, such stimulation reduced task-related prefrontal hyperactivity and normalized altered network configurations at during resting state. This study provides evidence that anodal tDCS could ameliorate cognitive dysfunction in MCI patients and temporarily reverse abnormalities in relevant brain activity.

#### Prefrontal Cortex

A recent study assessing MCI patients with Parkinson’s disease (PD; Manenti et al., 2016) evaluated the impact of up-regulating the right or left DLPFC via anodal tDCS combined with physical therapies. For 2 weeks, patients underwent daily administration of 2-mA stimulation during 25 min. Scores on the PD Cognitive Rating Scale and a verbal fluency test increased only in the real tDCS group (compared with the sham group). Notably, the effect remained stable for up to 3 months after the intervention.

#### Interim Discussion

These extremely preliminary results suggest that anodal tDCS can ameliorate cognitive dysfunction in different subtypes of MCI. In particular, long-term results obtained by combining stimulation with physical training open interesting avenues for further research.

## Challenges and Future Directions

So far, NIBS studies seeking to improve cognitive function in MCI/SCI patients have yielded inconclusive findings. Although some studies showed favorable results, statistically significant differences in an experimental setting do not necessarily imply clinical applicability. Consequently, the evidence gathered so far, though promising, must be considered extremely preliminary. No definite answer can yet be provided for the ultimate question underlying the field: can NIBS-induced cognitive improvements persist beyond treatment and translate into daily-life benefits? The methodological considerations listed below emerge as critical milestones to properly address this issue.

### Stimulation Parameters

A number of methodological considerations and caveats must be addressed before claiming that a genuine, reliable and replicable effect has been found. TMS stimulation protocols involve several parameters, such as stimulation frequency, intensity, coil shape, pulse and stimulation site. While neuroimaging tools have improved selection of target areas for stimulation, the latter are typically anatomically ill-defined and hugely variable among individual brains. This heterogeneity is reduced by using neuronavigation procedures and factoring individual anatomy into the protocol, thus maximizing the chances that one specific target node is being systematically stimulated within a critical network (Danner et al., 2008). Regarding stimulation frequency and intensity, a general finding is that TMS increases neuronal excitability above a 5-Hz frequency, whereas it yields the opposite effect below 1 Hz (Hallett, 2000; Sparing et al., 2001; Knoch et al., 2006). Stimulation intensity in TMS protocols is usually defined as a percentage of a subject’s individual motor threshold, namely, the minimal stimulation intensity that induces a reliable motor evoked potential of minimal amplitude in the targeted muscle (Rossini et al., 2015). This measure is useful to determine the intensity needed to comparably stimulate primary motor regions across subjects. However, the relevance of this parameter remains unknown for the stimulation of other cortical regions. Future research should examine the possibility of designing subject- and area-specific simulation protocols (Klooster et al., 2016), together with region-specific stimulation-tuning.

In the case of tDCS, key parameters include electrode patch positioning, inter-electrode distance, stimulation duration, and stimulation intensity (Klooster et al., 2016). With this technique, anodal and cathodal currents increase and inhibit neural-network activity, respectively. In this sense, one important difference with TMS is that tDCS allows stimulating and inhibiting different target regions at the same time (Ferrucci et al., 2009; Lindenberg et al., 2010; Mahmoudi et al., 2011), which increases the range of methodological decisions on the part of researchers. Another factor known to modulate tDCS results is age. Indeed, montages inducing effects in young adults do not necessary yield the same results in older adults (Zimerman et al., 2013). This highlights the importance of considering information about age-associated brain reorganization when aiming to induce durable neuroplastic effects in aging subjects (Perceval et al., 2016), including MCI patients.

### Behavioral Effects of Stimulation in Combination with Cognitive Training

Typically, neuro-rehabilitation protocols aim to stimulate the whole spatially-distributed network subserving a target cognitive function. To guarantee that the right mechanisms are being stimulated, brain enhancement must be combined with training of key relevant functions. Theoretically, targeting a dysfunctional neural circuit while it is actively engaged by a behavioral task should bring about better therapeutic effects than mere resting-state stimulation of the same area (Miniussi et al., 2013). Given that both cognitive training and NIBS can enhance adaptive plastic mechanisms, their combination could produce synergistic positive effects on behavior (Ditye et al., 2012). This approach offers a chance to stimulate very precise neurocognitive mechanisms, implement experimental designs with strict control of stimulus- and task-related factors and increase protocol viability through short-term longitudinal or pre/post models.

To date, this combined approach has been used in only one study (Park et al., 2014). After 10 daily sessions of tDCS with cognitive training, healthy elderly subjects increased working memory skills for up to 28 days. However, as this study lacked a control group without training, no further conclusions can be drawn regarding the potential benefits of combined NIBS-training interventions. Also relevant is the study protocol proposed by Cheng et al. (2015), aimed to improve working memory in aMCI patients. The design consists of a 4-week, double-blind, randomized controlled trial involving anodal tDCS or sham intervention combined with a working memory (*n*-back) paradigm or a control cognitive training condition. They hypothesize that this joint approach could significantly improve cognitive performance relative to isolated NIBS *or n-back* training. Furthermore, one of the studies reviewed above Manenti et al. (2016) reported cognitive enhancements in PD patients by combining tDCS with physical exercise cognitive enhancements. Taken together, this incipient evidence suggests that significant and long-lasting cognitive improvements could be induced through a combination of NIBS and specific cognitive training in MCI/SCI patients. Future research in the field should test this hypothesis by comparing the impact of joint and single-method interventions in both populations.

### The Critical Questions

#### Where to Stimulate?

Most of the reviewed studies targeted the DLPFC, a region implicated in working memory, everyday memory and automatic processing of learned tasks. These functions, indeed, were enhanced by NIBS in MCI patients and healthy subjects (Turriziani et al., 2012; Iuculano and Cohen Kadosh, 2013; Drumond Marra et al., 2015). Another region targeted within the frontal lobe has been the IFG. Stimulation over this structure significantly improved word retrieval and decreased hyperactivity in the bilateral PFC, the right middle temporal gyrus, the left basal ganglia, and the thalamus (Meinzer et al., 2015). Furthermore, rTMS over the STG has been observed to improve processing speed (Anderkova et al., 2015). Encouraging results have also been obtained through stimulation of the inferior parietal lobe, which enhanced associative and long-term memory in MCI patients (Cotelli et al., 2012). In this sense, note that other portions of the parietal cortex are known to increase blood oxygen level-dependent signals during working memory storage (Linden et al., 2003; Xu and Chun, 2006). The relevance of parietal structures as promising target areas for NIBS in MCI samples is highlighted by its role in cognitive impairments during early stage AD (Reiman et al., 2012; Weintraub et al., 2012).

**Figure 1:**
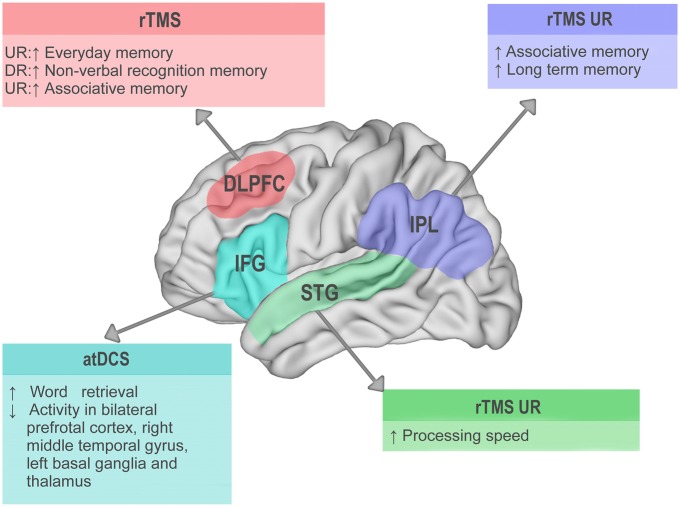
**Visual summary of main results from non-invasive brain stimulation (NIBS) studies with mild cognitive impairment (MCI) patients.** Lateral view of the left hemisphere. Target areas stimulated in the reviewed studies are highlighted in different colors. DLPFC, dorsolateral prefrontal cortex; IFG, inferior frontal gyrus; STG, superior temporal gyrus; IPL, inferior parietal lobule; atDCS, anodal transcranial direct current stimulation; rTMS, repetitive transcranial magnetic stimulation; ↑ behavioral improvement; ↓ behavioral decrement; UR, up-regulation; DR, down-regulation.

In sum, available evidence highlights the relevance of specific prefrontal, frontal, temporal, and parietal areas as key targets to develop more robust stimulation protocols in MCI. The main findings emerging from their stimulation in MCI patients are summarized in Figure 1.

#### How to Stimulate?

In general, rTMS protocols follow an offline approach, testing hypothesized cognitive changes roughly 30–60 min after stimulation (Ziemann et al., 2008). The use of patterned rTMS protocols, like theta burst stimulation, allows delivering 600 pulses with sub-threshold intensity in less than a minute. This induces longer-lasting effects in a shorter delivery time (Suppa et al., 2016). The impact of theta burst stimulation has been examined in several psychiatric conditions, like schizophrenia and obsessive-compulsive disorder (Suppa et al., 2016). However, studies in patients with memory impairment are still lacking.

On the other hand, tDCS is a smaller, portable tool which allows designing more ecological paradigms and obtaining online measurements. Thus, tDCS can be used to stimulate a particular neural network during behavioral intervention. Thanks to new developments, tDCS protocols can now employ more than two electrodes, enabling multifocal stimulation of brain networks to increase neurofunctional precision (Alam et al., 2016).

For increased effectiveness, NIBS can be applied in multi-session designs. Cognitive improvement in MCI patients is increased over time by repetitive, daily rTMS sessions (Cotelli et al., 2012; Drumond Marra et al., 2015). An alternative approach could rely on the use of spaced stimulation patterns, with multiple daily sessions. This might potentially lead to prolonged after effects via late-phase LTP/LTD-like neuroplastic mechanisms (Goldsworthy et al., 2015). Regarding tDCS, Manenti et al. (2016) worked with PD patients and found cognitive improvements lasting at least 3 months, after 10 daily sessions (Manenti et al., 2016). The only study employing tDCS in MCI patients employed a single-session protocol (Meinzer et al., 2015), so that no evidence is available on whether multiple-session tDCS protocols can induce long-term beneficial effects in this population.

Another important challenge lies in combining NIBS with daily life activities. This includes home-based training focused on specific cognitive skills. Self-delivered NIBS by the patient in a household environment, ideally according to a patient-specific stimulation protocol, may be a crucial step to hone the applicability of tDCS as a therapeutic tool (Klooster et al., 2016). To be effective, home-based portable devices should incorporate features which guarantee that stimulation is applied over the right brain regions, such as patient-specific cap-positioning tDCS electrodes. This may result in more efficient and less expensive cognitive intervention. In consequence, future research should develop new paradigms and technologies specific to each patient and relevant target areas.

#### Whom to Stimulate?

The use of NIBS to enhance memory in the elderly represents a promising avenue for both basic and translational research. However, the effects of NIBS likely depend on inter-individual differences in the degree of age-related cognitive impairment and brain reorganization (Craik, 1994; Mungas et al., 2010; Reuter-Lorenz and Park, 2014), under the influence of various genetic and lifestyle factors (Bishop et al., 2010; Grady, 2012). Moreover, different subtypes of MCI may respond in different ways to the same stimulation protocols. This calls for further research on the multiple subject-related variables that can modulate results within and across studies.

Correct diagnosis of MCI subtypes proves critical to discern stimulation effects. Approximately 50% of patients with aMCI convert to AD within 5 years (Gauthier et al., 2006). Also, different genetic influences impact on the development of AD. For instance, the presence of one or two ε4 alleles in the apolipoprotein E (APOE) gene confers risk of AD. An individual who meets the clinical, cognitive and etiologic criteria for MCI, and is also APOE ε4 positive, is more likely to progress to AD dementia within a few years than an individual without this genetic trait (Winblad et al., 2004; Anstey et al., 2008; Albert et al., 2011). Conversely, other familial versions will progress to AD with a 100% of probability (e.g., presenilin 1, PS1). Moreover, despite well-established risk genes (e.g., APOE, SORL1) or causative genes (e.g., APP, PSEN1, PSEN2) of AD, more than 20 loci have been associated with disease risk (Karch et al., 2014). In this sense, the epigenetic approach to clinical phenotypes offers a promissory agenda (Bennett et al., 2015). The need thus arises for more research on the interaction between NIBS and previous genetic risk in the MCI-AD spectrum.

On the other hand, fewer prognostic data are available about patients with non-memory-related impairments, who may develop non-AD dementia (Winblad et al., 2004; Anstey et al., 2008; Albert et al., 2011). Three out of the nine articles reviewed in “Does NIBS Improve Learning and Formation of Novel Memories in MCI?” Section targeted aMCI patients (Cotelli et al., 2012; Eliasova et al., 2014; Anderkova et al., 2015), one focused on vascular MCI (Sedlackova et al., 2008), and another one assessed MCI linked to PD (Manenti et al., 2016). The rest did not specify the MCI subtype. This distinction is crucial to assess the efficacy and extent of the stimulation protocol. For example, no effects were observed in vascular MCI when stimulating the DLPFC with rTMS (Sedlackova et al., 2008). It seems that each subtype of MCI could respond different to stimulation, although it is not clear which ones would profit the most from intervention. In consequence, it is necessary to stratify MCI subtypes to recognize condition-specific stimulation effects and develop more fine-grained protocols for each patient, according to his or her diagnosis.

Finally, given that aMCI patients may be at increased risk of developing AD, future interventions might focus on familial-MCI patients with high probability to progress into AD. Early-onset AD patients present MCI, rapidly followed by severe deficits in cortical functions (aphasia, apraxia, and agnosia), whereas late-onset AD patients show a slower overall decline and pronounced memory deficits, particularly in the semantic domain (Joubert et al., 2016). Since the progression of early-onset AD is faster than in late-onset AD, the latter variety may profit more from NIBS as a tool to attenuate the rate of cognitive decline. The identification of causative genetic mutations leading to early-onset AD (APP, PSEN1, PSEN2) and susceptibility markers associated with later disease onset (e.g., APOE ε4 carriers) is crucial to determine specific patterns of cognitive impairment in younger patients with a family history of AD or other neurodegenerative diseases (Rocchi et al., 2003; Winblad et al., 2004; Albert et al., 2011), and to better screen candidates for different stimulation protocols targeting specific neurofunctional mechanisms.

### A Call for Multicenter Studies

A thorough understanding of potential neurocognitive changes induced by NIBS requires protocols to be complemented with other functional techniques, like electroencephalography (EEG), magnetoelectroencephalography (MEG), positron-emission tomography (PET), fMRI, and magnetic resonance spectroscopy (MRS). Therefore, the direct comparison of neuro-anatomical changes before and after stimulation could shed light on the potentially specific effects of NIBS. Also, the establishment of NIBS techniques as therapeutic tools for MCI requires systematic assessment of multiple subject variables (e.g., cortical thinning, white matter volume and integrity, functional and structural connectivity, genetic variants, learning capacity, age at disease onset and cognitive reserve). In order to gain multidimensional insights into the impact of NIBS in MCI, above and beyond between-subject variability, longer multicenter studies are urgently needed. By revealing common patterns despite differences in diagnosis, sociodemographic factors, neurobiological features, brain recording parameters, measurements, and analyses, such joint protocols will help identify the most consistent changes induced by NIBS, while revealing the most effective stimulation parameters.

In this context, the implementation of normative databases based on neuroscientific techniques might help to recognize abnormal patterns, detect the disease at early stages, and define treatment protocol strategies (Gordon and Konopka, 2005). Available EEG neuromarkers of MCI and AD are critical to such ends (Ibanez and Parra, 2014). Quantitative EEG (qEEG) shows that AD and MCI are characterized by increased theta power, decreased alpha and beta power and decreased coherence in the alpha and theta band in posterior regions (Jelic et al., 2000). These abnormalities are thought to be associated with functional disconnections among cortical areas, loss of cortical neurons, axonal degeneration, and cholinergic deficits. Furthermore, EEG/MEG techniques have been useful to characterize AD and to detect changes in preclinical familial AD and MCI (Jackson and Snyder, 2008; Stam, 2010; Pietto et al., 2016). For example, source EEG functional network disruption in AD and MCI is associated with cognitive decline (Gianotti et al., 2007; Kurimoto et al., 2008; Ishii et al., 2010; Hsiao et al., 2013) and APOE genotype (Canuet et al., 2012). Moreover, relative to normative samples, subjects with SCI present elevated alpha power and increased number of spatiotemporal wave events (Alexander et al., 2006). Furthermore, event-related potential (ERP) measures have shown utility in predicting the conversion to dementia among elderly individuals at risk for AD (Gironell et al., 2005). Beyond its to diagnose functional impairment, EEG is also useful to set stimulation parameters and assess the effect of NIBS in patients (Kropotov, 2016). Combining NIBS with EEG would help to identify stimulus intensity, frequency of the stimulation, location, and duration needed to normalize EEG activity (Jin et al., 2012). This is critical since most rTMS protocols use the same parameters for all patients. With this approach, a customized treatment would be available for each individual based on analysis of resting EEG. In this sense, Marceglia et al. (2016) applied tDCS to change cortical activity as represented by qEEG. Despite working on a small sample, the authors found that the abnormal pattern of EEG activity in AD during memory processing is partially reversed by anodal transcranial direct current stimulation (atDCS) and this reversion correlated with an improvement in word recognition, suggesting that such benefits are supported by the modulation of neuronal cortical activity. In conclusion, normative databases should be systematically considered as a reference to establish the efficacy of individualized stimulation protocols.

## Conclusions

The present review aimed to summarize research on the effects of NIBS in MCI and to outline the approaches that may improve the efficacy of stimulation protocols. Despite major advances over the past few decades, extant MCI/AD treatments are not completely effective. Although NIBS have not yet been massively applied in SCI/MCI patients, available results hold considerable promise. First, TMS and tDCS are well-suited to explore brain plasticity across the lifespan (Heise et al., 2014). Second, they provide valuable physiologic biomarkers of the state of cortical reactivity, brain network connectivity, dynamics, and mechanisms of brain plasticity. Third, NIBS techniques avoid systemic side-effects and allow for temporally and spatially precise neural interventions that escape the possibilities of pharmacological or complementary therapies. Moreover, discrepancies among available studies may be largely due to differences among tasks, designs, and samples, as opposed to limitations inherent to NIBS. However, many issues remain to be solved and large longitudinal studies are needed to more stringently assess the robustness of available positive results. Furthermore, even if NIBS techniques proved irrelevant for clinical purposes, they could remain highly useful for experimental research. While no definitive conclusions on the efficacy and specificity of NIBS can be advanced at this stage, our review delineates the major challenges to be faced in future studies. In this sense, pre-post neuroimaging designs could reveal putative mechanisms underlying NIBS effects. Also, concurrent use of NIBS techniques with specific tasks targeting impaired domains in individual patients could potentially enhance LTP-like after-effects. In particular, applying NIBS over a dysfunctional neural network while the latter is engaged by a particular behavioral activity could significantly enhance learning- and memory-related effects. Furthermore, the application of NIBS across multiple sessions could render such effects longer lasting. All these considerations should be accompanied by very precise diagnosis of each patient’s MCI subtype, so that subject-specific protocols can be designed and assessed with the implementation of normative databases. Moreover, further studies are required to identify the optimal stimulation parameters yielding robust outcomes for different MCI subtypes as well as early- and late-onset AD. Finally, we emphasize the need to conduct longer longitudinal studies to establish the duration of NIBS-induced benefits. By contemplating these issues, future NIBS studies could shed unprecedented light on functional restoration options for MCI patients, paving the way for new treatments aimed to delay (or event prevent) the development of AD and other dementias.

## Author Contributions

AB did the literature research and wrote the manuscript. AI and AMG provided insightful thoughts to study concepts, and revised the manuscript. LS and JF assisted with the literature review and revised the final version. MZ wrote, revised critically and approved the final version.

## Conflict of Interest Statement

The authors declare that the research was conducted in the absence of any commercial or financial relationships that could be construed as a potential conflict of interest.
